# Patient‐reported evolution of the Cumberland Ankle Instability Tool after arthroscopic anatomic lateral ligament reconstruction: A retrospective study of 134 cases

**DOI:** 10.1002/jeo2.70952

**Published:** 2026-10-15

**Authors:** Ibrahim Saliba, Olivier Grimaud, Vincent Fontanier, Frederic Khiami, Yoann Bohu, Nicolas Lefevre, Alexandre Hardy

**Affiliations:** ^1^ Orthopedic Surgery Department Necker hospital Paris France; ^2^ Orthopedic Surgery Department Clinique Du Sport Paris France; ^3^ Medinetic Learning Paris France

**Keywords:** anatomic ankle lateral reconstruction, arthroscopy, CAIT, chronic lateral ankle instability, gracilis

## Abstract

**Purpose:**

To describe the longitudinal evolution of the Cumberland Ankle Instability Tool (CAIT) after arthroscopic anatomic lateral ligament reconstruction (AALR) and examine associations between CAIT‐defined patient‐perceived stability, return to sport (RTS) and preoperative factors.

**Methods:**

This retrospective single‐centre cohort included 134 athletic patients who underwent AALR between January 2019 and January 2023 and had at least 12 months of follow‐up. CAIT was assessed preoperatively and at 3, 6 and 12 months. Patient‐perceived stability was defined as CAIT ≥ 24. Repeated continuous and binary outcomes were assessed using paired‐data methods. RTS was defined as self‐reported resumption of sport by 12 months, irrespective of level.

**Results:**

Median CAIT increased from 8 preoperatively to 23, 26 and 28 at 3, 6 and 12 months, respectively (overall *p* < 0.001). CAIT‐defined stability increased from 5/134 (3.7%) preoperatively to 55/134 (41.0%), 91/134 (67.9%) and 96/134 (71.6%) at 3, 6 and 12 months (overall *p* < 0.001). Most improvement occurred by 6 months. Overall, 112 patients (83.6%) reported RTS by 12 months. Higher CAIT at 3, 6 and 12 months was associated with RTS. In exploratory multivariable analysis, preoperative ankle ligament reconstruction‐return to sport after injury was positively associated with 12‐month CAIT‐defined stability (odds ratio [OR]: 1.02, 95% confidence interval [CI]: 1.00–1.04; *p* = 0.043), whereas nonpivot sport was negatively associated compared with pivot‐contact sport (OR: 0.27, 95% CI: 0.09–0.75; *p* = 0.014). Four patients (3.0%) underwent fibular button removal.

**Conclusion:**

Patient‐reported ankle function measured by CAIT improved during the first postoperative year after AALR, with the largest change occurring by 6 months. CAIT may complement, but should not replace, objective examination and multidimensional RTS assessment. The identified associations are exploratory and require prospective validation.

**Level of Evidence:**

Level III.

**Abbreviations**
AALR: arthroscopic anatomic lateral ligament reconstruction
ALR‐RSI: ankle ligament reconstruction‐return to sport after injury
ATFL: anterior talofibular ligament
BMI: body mass index
CAI: chronic ankle instability
CAIT: Cumberland Ankle Instability Tool
CFL: CalcaneoFibular Ligament
CI: confidence interval
FAAM: foot and ankle ability measure
FAOS: foot and ankle outcome score
IAC: International Ankle Consortium
OR: odds ratio
PROM: patient‐reported outcome measure
RTS: return to sport

## INTRODUCTION

Chronic ankle instability (CAI) is a common consequence of recurrent ankle sprain and may require surgery when structured nonoperative treatment fails [17, 21]. Anatomical lateral ligament reconstruction aims to restore ligament orientation and may be considered when direct repair is unsuitable or at increased risk of failure [1, 8, 18, 19, 20, 26, 29, 30].

Patient‐reported outcome measures (PROMs) capture symptoms and functional limitations from the patient's perspective [11, 14, 32]. The Cumberland Ankle Instability Tool (CAIT) is a concise nine‐item questionnaire scored from 0 to 30, with lower scores reflecting greater patient‐perceived functional instability [10]. It has demonstrated validity and reliability in several languages [4, 6, 10, 13, 22, 33, 34]. The International Ankle Consortium proposed a threshold of <24 to identify individuals with CAI in research settings [7]. A previous study from our group compared CAIT with foot and ankle ability measure (FAAM) for discrimination of return to sport (RTS) status and provided the rationale for longitudinal assessment of CAIT; those findings are not outcomes of the present analysis [24]. Importantly, CAIT is a PROM and does not directly quantify mechanical ligament laxity.

The primary objective was to describe changes in CAIT scores and the proportion of patients reaching CAIT ≥ 24 during the first postoperative year after arthroscopic anatomic lateral ligament reconstruction (AALR). Secondary objectives were to examine the association of CAIT scores and CAIT‐defined patient‐perceived stability with self‐reported RTS and to explore preoperative factors associated with CAIT‐defined stability at 12 months. We did not aim to compare AALR with another treatment or to establish predictors of RTS.

## MATERIALS AND METHODS

This retrospective analysis of a prospectively maintained institutional ankle registry (Ankle Clinique Du Sport) was approved by the Institutional Review Board of the study centre [Conseil d'Orientation Scientifique Ramsay Santé IRB00010835; approval number: COS‐RGDS‐2025‐07‐005‐HARDY‐A].

All patients provided electronic informed consent for inclusion in the registry and secondary use of anonymised data for research. The registry is identified as NCT07188363; this identifier refers to the parent registry and not to prospective registration of the present retrospective analysis. The study was reported in accordance with the strengthening the reporting of observational studies in epidemiology statement, and a completed checklist is supplied as Supporting Information.

### Study population

The study population comprised athletic patients recorded in the institutional ankle registry who underwent AALR at a specialised sports surgery centre between January 2019 and January 2023. Patients were eligible if they had at least 12 months of follow‐up. Exclusion criteria were follow‐up shorter than 12 months, lower‐extremity surgery during the 2 years preceding AALR, concomitant talar or tibial cartilage lesions, varus hindfoot alignment or radiographic ankle osteoarthritis. The final study sample comprised 134 patients. Because this was a retrospective study of all eligible registry patients, no prospective sample‐size calculation was performed.

### Indication for surgery

Surgery was indicated for recurrent ankle sprains, giving way or persistent perceived instability despite at least 6 months of nonoperative management [3, 25, 27, 28]. A positive talar tilt test was required as clinical evidence of mechanical instability. Patients with varus hindfoot alignment or radiographic ankle osteoarthritis were not included.

### Surgical procedure

All patients underwent the same arthroscopic AALR technique [17]. The anterior talofibular ligament and calcaneofibular ligament were reconstructed with an ipsilateral gracilis autograft soaked in vancomycin before fixation [23].

Under arthroscopic guidance, the graft was fixed in a 5.0‐mm blind talar tunnel and a 5.5‐mm calcaneal tunnel. Proximal fixation was achieved through a 6.0‐mm fibular tunnel using cortical suspension.

Immediate weight‐bearing was allowed in a walking boot for 3 weeks, followed by a standardised rehabilitation program emphasising strength, balance and proprioception. Ankle‐GO was used as one component of the RTS assessment; return to the preinjury level was permitted when the Ankle‐GO score exceeded 14 [9].

### Outcome measures

The primary outcome was the CAIT score at baseline and 3, 6 and 12 months [10]. CAIT ranges from 0 (greatest patient‐perceived instability) to 30 (least patient‐perceived instability). In accordance with the International Ankle Consortium threshold, CAIT ≥ 24 was categorised as CAIT‐defined patient‐perceived stability [7]. This designation refers to a questionnaire threshold and not to an objective measure of ligament laxity.

The principal secondary outcome was self‐reported RTS by 12 months. RTS was analysed as a binary response (yes/no) and therefore represents return to participation in any sport, irrespective of return to the preinjury level, performance, exact timing or sustained participation. Associations of RTS with CAIT scores and CAIT‐defined stability were examined. Baseline age, sex, body mass index (BMI), operated side, arthroscopic injury stage, preinjury practice level, sport category, CAIT, ankle ligament reconstruction‐return to sport after injury (ALR‐RSI), FAOS and FAAM were compared between RTS and no‐RTS groups. Foot and ankle outcome score (FAOS) and FAAM were collected longitudinally in the registry, but their postoperative trajectories were outside the prespecified scope of this CAIT‐focused analysis; baseline values were considered only as candidate covariates.

Postoperative complications were reviewed, including surgical‐site infection, nerve injury, graft failure or recurrent instability, fixation‐related symptoms, reoperation and revision surgery.

### Data collection

Data were obtained from a prospectively maintained web‐based institutional registry. Eligible patients were identified using diagnosis and procedure fields corresponding to AALR. Data were analysed retrospectively.

Patients completed CAIT, ALR‐RSI, FAOS and FAAM electronically before surgery. Follow‐up questionnaires were scheduled at 3, 6 and 12 months. Automated email and text‐message reminders were sent for missing questionnaires, and completion was encouraged during clinical follow‐up. Analyses used available observations; denominators are reported where data were missing. The anonymised individual‐level dataset used for this study is provided as Supplementary Material.

### Participants

A total of 134 patients met the eligibility criteria and were included. The cohort comprised 64 men and 70 women, with a median age of 29 years (IQR: 24–39). The derivation of the cohort and available data at each assessment are described in the text and tables.

### Statistical analysis

Analyses were conducted using R version 3.5.0 (R Foundation for Statistical Computing).

Continuous variables are reported as median and interquartile range (IQR), and categorical variables as number and percentage. The overall change in repeated CAIT scores across baseline, 3, 6 and 12 months was assessed using the Friedman test. Pairwise within‐patient comparisons were performed using Wilcoxon signed‐rank tests with Holm adjustment. Change in the repeated binary outcome CAIT ≥ 24 was assessed using Cochran's *Q*‐test, followed by paired McNemar comparisons with Holm adjustment. Comparisons between the independent RTS and no‐RTS groups used the Wilcoxon rank‐sum test for continuous variables and Pearson chi‐square or Fisher exact tests for categorical variables, as appropriate. All tests were two‐sided, with *p* < 0.05 considered statistically significant.

The association between CAIT‐defined stability and RTS was examined at each postoperative assessment. Because RTS was recorded as a binary 12‐month endpoint, these analyses describe association and do not establish temporal causation or prediction.

An exploratory multivariable logistic regression evaluated factors associated with CAIT‐defined stability at 12 months. Candidate variables were prespecified on clinical grounds and reviewed in univariable analyses; to limit overfitting, the final model was restricted to preoperative ALR‐RSI and sport category. Pivot‐contact sport was the reference category. Adjusted odds ratios (ORs) with 95% confidence intervals (CIs) are reported. No internal or external model validation was performed; accordingly, the model is interpreted as an exploratory association rather than a validated prediction model.

## RESULTS

### Patient characteristics

The 134 patients comprised 64 men (47.8%) and 70 women (52.2%). Median age was 29 years (IQR: 24–39) and median follow‐up was 16 months. Baseline characteristics and available denominators are summarised in Table 1.

**Table 1:** Patient characteristics and comparison between RTS and No‐RTS groups.

Characteristic	Description of patients with AALR who have returned to sport or not			*p*‐Value
	No RTS *n* = 22	RTS *n* = 112	Overall *n* = 134	
Age (years), Median (25%–75%)	29 (25–40)	29 (23–39)	29 (24–39)	0.890^a^
Sex, *n* (%)				0.482^b^
Female	13 (59.1)	57 (50.9)	70 (52.2)	
Male	9 (40.9)	55 (49.1)	64 (47.8)	
Operated side, *n* (%)				0.304^b^
Right	12 (54.5)	71 (63.4)	83 (62.0)	
Left	10 (45.5)	41 (36.6)	51 (38.0)	
BMI (kg/m^2^), *n* (%)				0.558^c^
Underweight (<18.5)	3 (13.6)	7 (6.3)	10 (7.5)	
Normal (18.5–24.9)	10 (45.5)	63 (56.3)	73 (54.5)	
Overweight (25.0–29.9)	4 (18.2)	32 (28.6)	36 (26.9)	
Obesity (>30)	5 (22.7)	10 (8.9)	15 (11.2)	
Arthroscopic injury stage, *n* (%) [available *n* = 118]				>0.999^c^
0: normal ATFL thickness and tension	0 (0.0)	0 (0.0)	0 (0.0)	
1: ATFL distension with normal thickness	0 (0.0)	3 (3.1)	3 (2.5)	
2: ATFL avulsion with normal thickness	8 (40.0)	37 (37.8)	45 (38.1)	
3: thin ATFL with no resistance during the hook test	5 (25.0)	26 (26.5)	31 (26.3)	
4: no ATFL, with a bald malleolus	7 (35.0)	32 (32.7)	39 (33.1)	
Level of practice, *n* (%)				**0.015** c
Competition	5 (22.7)	45 (40.2)	50 (37.3)	
Occasional leisure	8 (36.4)	14 (12.5)	22 (16.4)	
Regular leisure	9 (40.9)	35 (31.3)	44 (32.8)	
Professional	0 (0.0)	18 (16.1)	18 (13.4)	
Type of sport, *n* (%)				**<0.001** c
Nonpivot, noncontact	7 (31.8)	35 (31.3)	42 (31.3)	
Pivot	11 (50.0)	28 (25.0)	39 (29.1)	
Pivot‐contact	4 (18.2)	49 (43.8)	53 (39.6)	
Preoperative CAIT, median (25%–75%)	7 (6–9)	8 (6–13)	8 (6–12)	0.147^a^
3 months CAIT, median (25%–75%)	12 (10–22)	23 (20–27)	23 (18–26)	**0.013** a
6 months CAIT, median (25%–75%)	18 (11–24)	27 (24–30)	26 (21–29)	**<0.001** a
12 months CAIT, median (25%–75%)	18 (12–24)	28 (25–30)	28 (19–29)	**<0.001** a

*Note*: Bold values are statistically significant at *p* < 0.05.

Abbreviations: AALR, arthroscopic anatomic lateral ligament reconstruction; ATFL anterior talofibular ligament; BMI, body mass index; CAIT, Cumberland Ankle Instability Tool; RTS, return to sport.

^a^ Wilcoxon rank sum test.

^b^ Pearson's Chi‐squared test.

^c^ Fisher's exact test.

### Evolution of CAIT scores

Median CAIT increased from 8 (IQR: 6–12) preoperatively to 23 (IQR: 18–26) at 3 months, 26 (IQR: 21–29) at 6 months and 28 (IQR: 19–29) at 12 months (overall Friedman *p* < 0.001).

Holm‐adjusted paired comparisons showed improvement from baseline to each postoperative assessment (all *p* < 0.001), from 3 to 6 months (*p* < 0.01) and from 3 to 12 months (*p* < 0.001). The difference between 6 and 12 months was not statistically significant (*p* = 0.37) (Figure 1).

**Figure 1:**
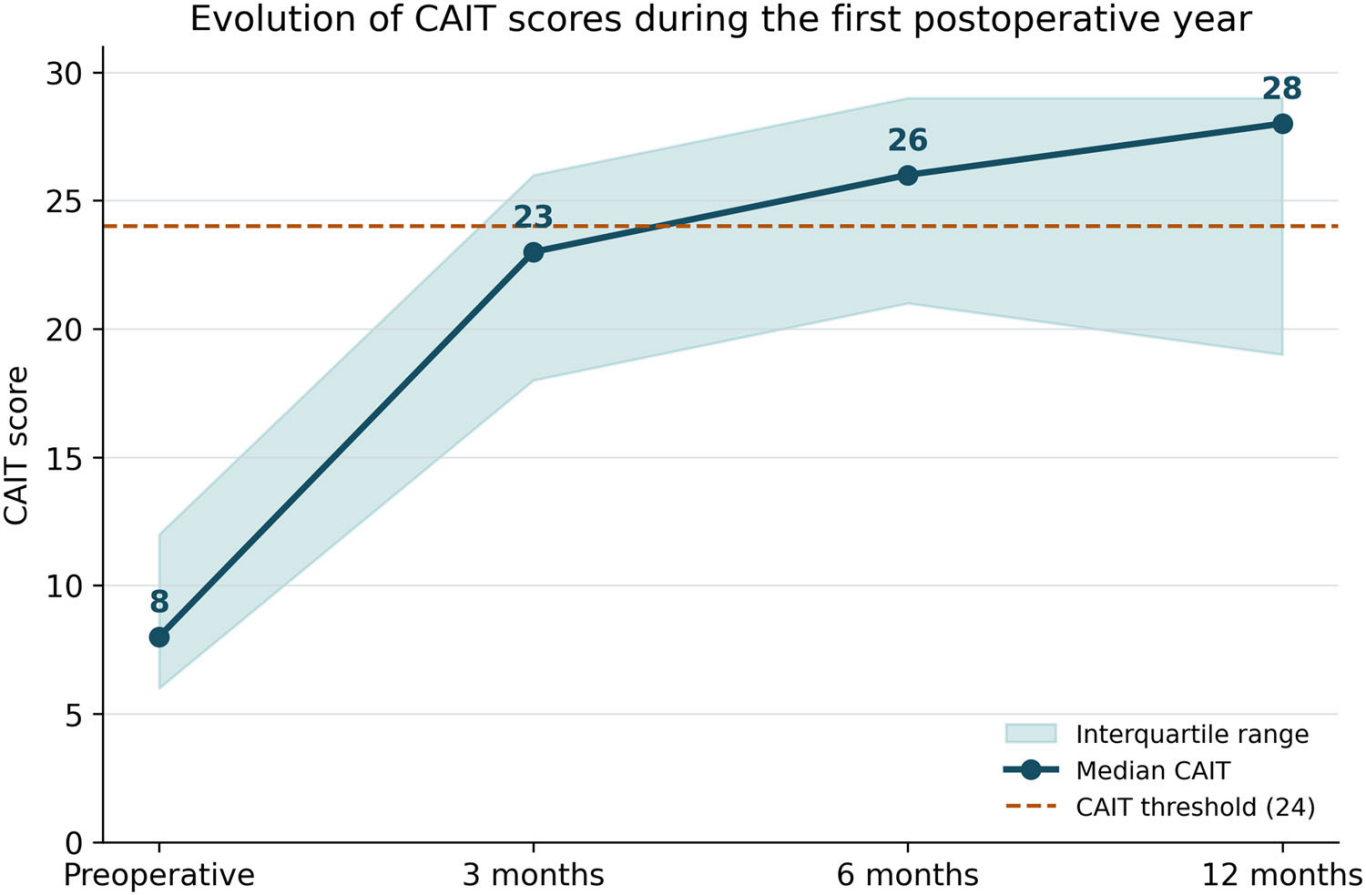
Evolution of CAIT scores during the first postoperative year. The line shows reported medians, and the shaded area shows interquartile ranges. The dashed line denotes the CAIT threshold of 24. CAIT is a patient‐reported measure and does not directly assess mechanical laxity. CAIT, Cumberland Ankle Instability Tool.

### Evolution of CAIT‐defined patient‐perceived stability

The proportion with CAIT ≥ 24 increased from 5/134 (3.7%) preoperatively to 55/134 (41.0%) at 3 months, 91/134 (67.9%) at 6 months and 96/134 (71.6%) at 12 months (overall Cochran's *Q p* < 0.001) (Table 2).

**Table 2:** Evolution of CAIT‐defined patient‐perceived stability (CAIT ≥ 24). The proportion of patients with CAIT ≥ 24 increased over time (Cochran's Q *p* < 0.001).

Timepoint	Stable *n* (%)	Unstable *n* (%)
Preoperative	5 (3.7)	129 (96.3)
3 months	55 (41.0)	79 (59.0)
6 months	91 (67.9)	43 (32.1)
12 months	96 (71.6)	38 (28.4)

Abbreviation: CAIT, Cumberland Ankle Instability Tool.

Adjusted paired comparisons showed significant increases through 6 months, whereas the change between 6 and 12 months was not statistically significant. Because CAIT is patient‐reported, these proportions describe CAIT‐defined perceived stability rather than mechanical ligament stability.

### RTS and association with stability

Overall, 112 patients (83.6%) reported resuming sport by 12 months; 22 (16.4%) did not. This endpoint indicates return to participation and does not distinguish preinjury level, performance, timing or sustained participation. CAIT scores were higher among patients who reported RTS (Table 1).

Median CAIT at 3 months was 23 in the RTS group and 12 in the no‐RTS group (*p* = 0.013). Corresponding values were 27 and 18 at 6 months (*p* < 0.001) and 28 and 18 at 12 months (*p* < 0.001). Baseline CAIT did not differ significantly (*p* = 0.147).

CAIT‐defined stability at 3 and 6 months was associated with 12‐month RTS status (*p* = 0.003 and *p* < 0.001, respectively); the association at 12 months was not statistically significant. These unadjusted associations should not be interpreted as evidence that early CAIT status causes RTS.

### Factors associated with CAIT‐defined stability at 12 months

In the exploratory multivariable model, each one‐point increase in preoperative ALR‐RSI was associated with an adjusted OR of 1.02 for CAIT‐defined stability at 12 months (95% CI: 1.00–1.04; *p* = 0.043) (Table 3; Figure 2). The effect estimate was close to the null and should be interpreted cautiously.

**Table 3:** Exploratory multivariable analysis of factors associated with CAIT‐defined patient‐perceived stability (CAIT ≥ 24) at 12 months.

Variable	Odds ratio (95% CI)	*p*‐Value
Preoperative ALR‐RSI (per 1‐point increase)	1.02 (1.00–1.04)	0.043
Type of sport		
Pivot‐contact (reference)	—	—
Pivot	0.35 (0.11–0.99)	0.051
Nonpivot (line)	0.27 (0.09–0.75)	0.014

*Note*: Multivariable logistic regression of factors associated with CAIT‐defined patient‐perceived stability at 12 months. Odds ratios (OR) with 95% confidence intervals (CI) are reported. Pivot‐contact sports were used as the reference category.

Abbreviations: ALR‐RSI, ankle ligament reconstruction‐return to sport after injury; CAIT, Cumberland Ankle Instability Tool.

**Figure 2:**
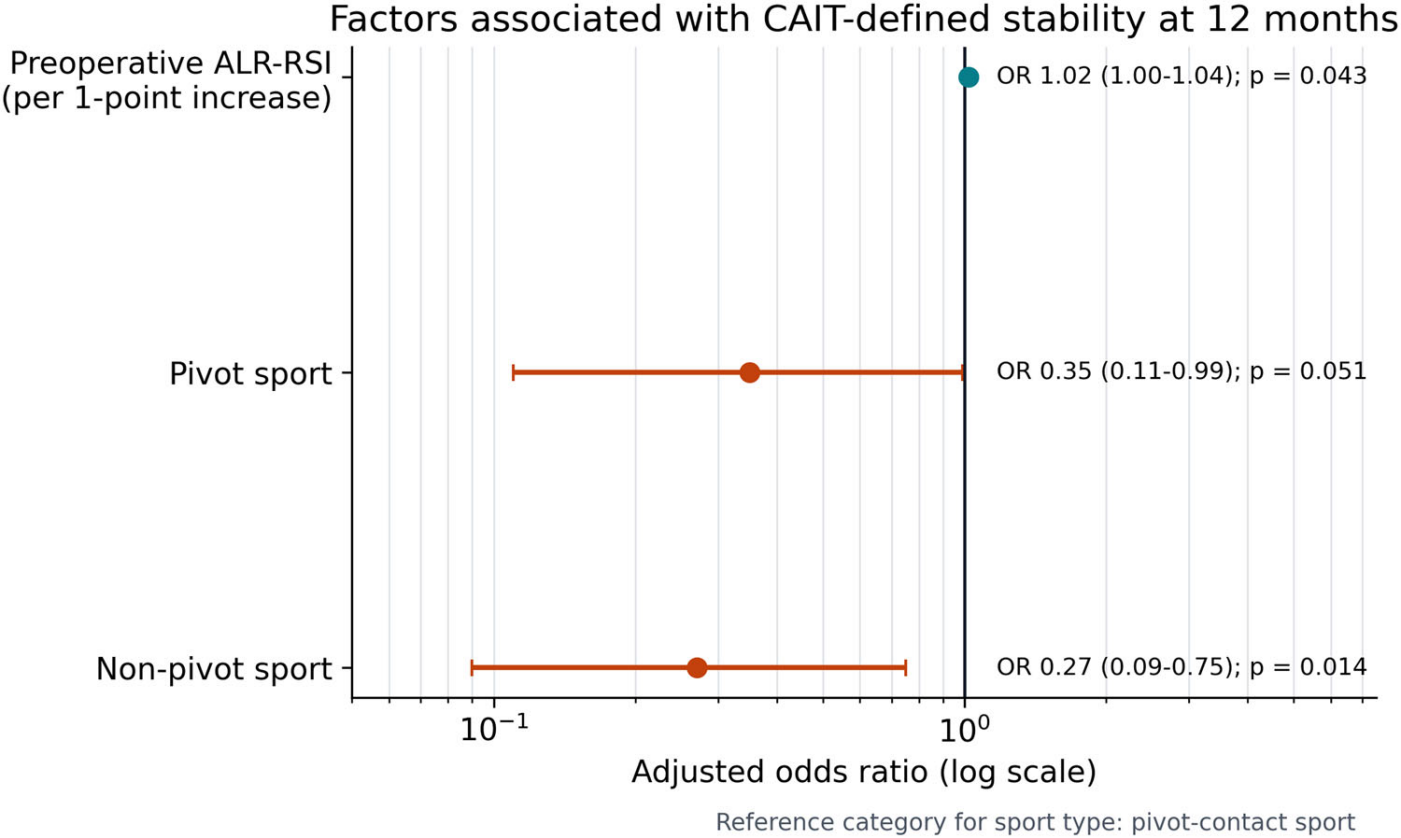
Factors associated with CAIT‐defined patient‐perceived stability at 12 months. Forest plot of adjusted odds ratios and 95% confidence intervals from the exploratory logistic regression. Pivot‐contact sport is the reference category. Estimates are exploratory and have not undergone internal or external model validation. ALR‐RSI, ankle ligament reconstruction‐return to sport after injury; CAIT, Cumberland Ankle Instability Tool; OR, odds ratio.

Using pivot‐contact sport as the reference, nonpivot sport was associated with lower odds of CAIT‐defined stability (OR: 0.27, 95% CI: 0.09–0.75; *p* = 0.014). The estimate for pivot sport was OR: 0.35 (95% CI: 0.11–0.99; *p* = 0.051). These associations are exploratory and may reflect residual confounding.

Age, BMI and arthroscopic injury stage were not significantly associated with CAIT‐defined stability in univariable analyses.

### Complications

No surgical‐site infection, nerve injury, documented graft failure or revision ligament reconstruction was identified during the available follow‐up. Four patients (3.0%) underwent secondary removal of a symptomatic fibular cortical button within 6 months.

## DISCUSSION

The principal finding was a marked improvement in patient‐reported ankle function measured by CAIT during the first postoperative year after AALR. The largest change occurred between baseline and 3 months, with smaller changes thereafter and no statistically significant difference between 6 and 12 months. Because this was an uncontrolled retrospective study, the observed trajectory cannot be attributed solely to the surgical technique; rehabilitation, recovery over time, patient selection and other unmeasured factors may have contributed.

The temporal pattern is compatible with published RTS intervals after lateral ligament repair or reconstruction, which commonly range from approximately 3 to 6 months [2, 9, 12, 15]. Nevertheless, the present study did not compare AALR with Brostrom repair, open reconstruction, continued nonoperative treatment or another rehabilitation strategy. It therefore documents postoperative change within this cohort, and neither demonstrates superiority nor provides a comparative estimate of effectiveness.

Analysing CAIT both continuously and using the threshold of 24 facilitates clinical interpretation. However, the threshold originates from research classification of CAI and should not be considered a direct test of reconstructed ligament integrity [7, 10, 16, 33]. CAIT captures patient‐perceived functional instability; stress radiographs, instrumented laxity testing, clinical examination or imaging would be required to assess mechanical stability. We therefore revised the terminology throughout to ‘CAIT‐defined patient‐perceived stability’.

Higher CAIT at 3 and 6 months was associated with self‐reported RTS by 12 months. This finding may help identify patients who report persistent functional difficulty during rehabilitation, but it does not establish a causal pathway or a stand‐alone RTS threshold. RTS decisions are multidimensional and should incorporate symptoms, objective function, sport‐specific testing, psychological readiness, clinician assessment and exposure to progressive training.

Preoperative ALR‐RSI and sport category were associated with 12‐month CAIT‐defined stability in an exploratory model. The ALR‐RSI effect was small per scale point, and its CI approached the null. The lower odds observed for nonpivot sport relative to pivot‐contact sport were not hypothesised as a protective effect and may reflect differences in conditioning, rehabilitation exposure, expectations or unmeasured case mix. These results should be viewed as hypothesis‐generating rather than as validated predictors.

The binary RTS rate of 83.6% provides only a broad measure of participation. Return to participation, return to the preinjury level, return to performance, time to RTS and sustained participation were not distinguished. Consequently, this study cannot determine whether patients regained their prior competitive level or performance, and the RTS analyses should be interpreted within that limitation.

Age and BMI were not significantly associated with RTS in this cohort, although prior work has suggested small adverse associations with increasing age and BMI [15]. The selected athletic population and the limited number of patients in some subgroups may have reduced the ability to detect such associations.

The arthroscopic injury stage was not associated with RTS. Although anatomical reconstruction may reduce dependence on residual ligament quality, the present study was not designed to establish equivalence across injury stages, and the finding may reflect limited power or residual confounding [5, 31].

Four patients underwent removal of a symptomatic fibular button. No other major event was identified in the registry during the available follow‐up. Because complications were assessed retrospectively and follow‐up was relatively short, uncommon or delayed events may have been missed; the data do not support a definitive claim of procedural safety or graft durability.

CAIT is concise and has strong measurement properties across several language versions [4, 6, 10, 13, 22, 33, 34]. In this setting, it may be useful for monitoring patient‐perceived functional instability alongside other clinical and functional assessments. Reference [24] supports the rationale for studying CAIT relative to FAAM; the present longitudinal results are distinct and are not derived from that publication.

This study has important limitations. Its retrospective, uncontrolled design is susceptible to selection bias, information bias and uncontrolled confounding and precludes causal inference. There was no comparison group, and all patients were treated at a single specialised sports centre with one technique; exclusions of previous surgery, cartilage lesions, deformity and osteoarthritis limit generalisability. Rehabilitation adherence and intensity, generalised ligamentous laxity, smoking, comorbidities, occupational demands, chronicity, graft characteristics, surgeon effects and additional psychological factors were not available. RTS was binary and did not capture level, performance, timing or durability. CAIT is a PROM rather than a mechanical‐stability measure, and no objective laxity assessment was analysed. The median follow‐up of 16 months is insufficient to evaluate recurrent instability, graft durability, osteoarthritis or sustained sport participation. No a priori sample‐size calculation was performed, missing data may have affected some analyses and the multivariable model was not validated. These limitations require cautious interpretation and prospective multicenter confirmation.

## CONCLUSION

During the first postoperative year after AALR, this cohort showed substantial improvement in patient‐reported functional ankle stability measured by CAIT, with the largest change occurring by 6 months. Higher postoperative CAIT was associated with self‐reported RTS, and preoperative ALR‐RSI and sport category showed exploratory associations with 12‐month CAIT‐defined stability. Because the study was retrospective and uncontrolled, these findings describe postoperative trajectories and associations rather than causal effectiveness, mechanical stability or superiority. CAIT may complement, but should not replace, objective examination and multidimensional RTS assessment.

## AUTHOR CONTRIBUTIONS

**Ibrahim Saliba**: contributed to the study design and article writing. **Olivier Grimaud**: participated in data collection. **Vincent Fontanier**: participated in data analysis. **Frederic Khiami**: participated in data collection and provided critical feedback. **Yoann Bohu**: participated in data collection and provided critical feedback. **Nicolas Lefevre**: provided critical feedback. **Alexandre Hardy**: contributed to the study design, article writing and critical feedback.

## FUNDING INFORMATION

The authors have no funding to report.

## CONFLICT OF INTEREST STATEMENT

The authors declare no conflicts of interest.

## ETHICS STATEMENT

This study was approved by the Institutional Review Board of the study centre [Conseil d'Orientation Scientifique Ramsay Santé IRB00010835; approval number: COS‐RGDS‐2025‐07‐005‐HARDY‐A]. Informed consent was obtained from all the patients.

## Supporting information

Supplementary Dataset. Anonymized individual‐level study dataset, including demographic, clinical, surgical and follow‐up variables used in the analyses.

CAIT_STROBE_checklist.

## Data Availability

The data that support the findings of this study are available in the supplementary material of this article.
