# Supplementary material for: Patient‐reported evolution of the Cumberland Ankle Instability Tool after arthroscopic anatomic lateral ligament reconstruction: A retrospective study of 134 cases
Source: J Exp Orthop. 2026 Oct 15;13(4):e70952. doi: 10.1002/jeo2.70952 (PMC13671355; doi:10.1002/jeo2.70952)
Supplement: Supplementary file 1 [file JEO2-13-e70952-s001.docx]

STROBE Checklist for Cohort Studies

Manuscript: Patient-Reported Evolution of the CAIT After Arthroscopic AALR

| **Item** | **Recommendation** | **How addressed** | **Location** |
| --- | --- | --- | --- |
| 1(a) | Title and abstract | Study design indicated in title; structured abstract summarizes design and results. | Title; Abstract |
| 1(b) | Abstract | Balanced summary of methods and findings. | Abstract |
| 2 | Background/rationale | Scientific background and CAIT rationale. | Introduction, paragraphs 1-2 |
| 3 | Objectives | Primary and secondary objectives stated. | Introduction, final paragraph |
| 4 | Study design | Retrospective analysis of a prospectively maintained registry. | Materials and methods, opening paragraph |
| 5 | Setting | Specialized sports surgery center; dates and follow-up reported. | Study population |
| 6(a) | Participants | Eligibility and exclusion criteria described. | Study population |
| 6(b) | Matching | Not applicable; no matched groups. | Not applicable |
| 7 | Variables | CAIT, CAIT >=24, RTS, covariates, and complications defined. | Outcome measures |
| 8 | Data sources/measurement | Electronic registry and scheduled questionnaires described. | Data collection |
| 9 | Bias | Selection, information, and confounding discussed. | Discussion, Limitations |
| 10 | Study size | All eligible patients; no prospective calculation. | Study population |
| 11 | Quantitative variables | CAIT scale and threshold; BMI and sport categories described. | Outcome measures; Table 1 |
| 12(a) | Statistical methods | Repeated-measures, group comparisons, and logistic model described. | Statistical analysis |
| 12(b) | Subgroups/interactions | RTS/no-RTS subgroup comparisons described. | Statistical analysis; Table 1 |
| 12(c) | Missing data | Available-observation approach and denominators noted. | Data collection; Table 1 |
| 12(d) | Loss to follow-up | Minimum follow-up criterion specified. | Study population |
| 12(e) | Sensitivity analyses | No sensitivity analysis performed. | Limitations |
| 13(a-c) | Participants | Included sample and available data described; reasons for exclusion specified. | Study population; Participants |
| 14(a-c) | Descriptive data | Demographic, clinical, and missing-variable denominators reported. | Results; Table 1 |
| 15 | Outcome data | CAIT trajectories, CAIT >=24, RTS, and complications reported. | Results; Tables 2-3 |
| 16(a-c) | Main results | Unadjusted comparisons and adjusted ORs with CIs reported. | Results; Tables 1-2 |
| 17 | Other analyses | Pairwise repeated-measures analyses reported. | Evolution of CAIT scores |
| 18 | Key results | Principal findings summarized relative to objectives. | Discussion, opening paragraph |
| 19 | Limitations | Bias, confounding, precision, follow-up, and generalizability discussed. | Discussion, Limitations |
| 20 | Interpretation | Cautious interpretation considering design and evidence. | Discussion; Conclusion |
| 21 | Generalisability | Single-center selected athletic cohort addressed. | Discussion, Limitations |
| 22 | Funding | To be completed in the journal declarations form/title page. | Declarations/title page |
